# Detecting the Hydrogen Bond Cooperativity in a Protein β-Sheet by H/D Exchange

**DOI:** 10.3390/ijms232314821

**Published:** 2022-11-26

**Authors:** Jingwen Li, Jingfei Chen, Yefei Wang, Lishan Yao

**Affiliations:** 1 College of Chemistry and Chemical Engineering, China University of Petroleum (East China), Qingdao 266580, China; 2 Qingdao New Energy Shandong Laboratory, Qingdao Institute of Bioenergy and Bioprocess Technology, Chinese Academy of Sciences, Qingdao 266101, China; 3 Shandong Energy Institute, Qingdao 266101, China

**Keywords:** H-bond cooperativity, β-sheet, NMR, H/D exchange

## Abstract

The hydrogen bond (H-bond) cooperativity in the β-sheet of GB3 is investigated by a NMR hydrogen/deuterium (H/D) exchange method. It is shown that the weakening of one backbone N–H…O=C H-bond between two β-strands, β1 and β2, due to the exchange of NH to ND of the H-bond donor in β1, perturbs the chemical shift of ^13^C_α_, ^13^C_β_, ^1^H_α_, ^1^H_N_, and ^15^N of the H-bond acceptor and its following residue in β2. Quantum mechanical calculations suggest that the -H-bond chemical shift isotope effect is caused by the structural reorganization in response to the H-bond weakening. This structural reorganization perturbs four neighboring H-bonds, with three being weaker and one being stronger, indicating that three H-bonds are cooperative and one is anticooperative with the perturbed H-bond. The sign of the cooperativity depends on the relative position of the H-bonds. This H-bond cooperativity, which contributes to β-sheet stability overall, can be important for conformational coupling across the β-sheet.

## 1. Introduction

Cooperativity has been widely observed in biologic processes such as protein-ligand binding [1], protein folding [2,3], and enzyme catalysis [4,5]. This cooperativity originates from the atomic interaction network, which is crucial for maintaining the structure and function of biomolecules [6,7]. Hydrogen bonding (H-bonding) is one of the most important noncovalent interactions in proteins. It is generally agreed that H-bonds are cooperative in protein α-helices. This is due to the α-helix’s electric dipole moment, which decreases hydrogen-bonding and long-range electrostatic interactions between backbone amides [8,9,10]. However, in β-sheets different conclusions have been drawn from computational studies. Although it is generally accepted that stronger backbone hydrogen bonds can form with more sheets, the existence of H-bond cooperativity along the strand direction is controversial. And it is still unclear whether the cooperativity originates from electrostatic polarization, elastic processes, or a combination of both [11,12,13,14]. In addition, elucidating the H-bond cooperativity in β-sheets may aid in the understanding of important process involving this secondary structure motif, such as amyloid fibril formation [15,16,17] and self-assembly [18,19]. Therefore, direct experimental characterization of H-bond cooperativity in β-sheets is of great theoretical and practical significance, but remains challenging.

Until now, most of the literature on protein H-bond cooperativity has relied on computational simulations or experimental studies employing protein secondary structure analogues [20,21]. Nuclear magnetic resonance (NMR) is a powerful tool in studying H-bonds in biomolecules [2,6,22,23,24,25,26,27]. Using parameters such as chemical shifts [28,29], J-coupling constants [30,31,32], and H-D exchange rates [33,34], it is possible to study not only individual hydrogen bonds’ strengths but also their couplings. In conjunction with computational simulations, ^1^H NMR can reveal the H-bonding coupling within the active site of some enzymes, such as ketosteroid isomerase and photoactive yellow protein [35,36]. In addition, the J-coupling constants such as *^3h^J_NC_′* and *^1^J_NC_′* can be used to quantify the strength of protein backbone H-bonds as well as the coupling between adjacent peptide planes [32,37]. H/D exchange is routinely used to study protein local conformational changes [38,39]. At the so-called EX1 limit, the measured H/D exchange rate corresponds to the opening rate of local conformation. At the so-called EX2 limit, the rate is proportional to the open conformational population. Moreover, the isotope effect generated by H/D exchange can influence the chemical shifts of nearby atoms, revealing relevant hydrogen bond information [28,33]. However, the β-sheet H-bond cooperativity has yet to be quantitatively characterized by systematic experimental evidence.

In this work, we use the protein GB3 (56 amino acids, the third IgG-binding domain from Streptococcal protein-G) [40] as a model system and investigate the β-sheet H-bond cooperativity through the H/D isotope effect [10] obtained from the NMR H/D exchange measurement [41]. Due to the Ubbelohde effect [42], the substitution of ^1^H by ^2^H in a backbone N-H…O=C H-bond weakens the H-bond’s (primary H-bond) strength [43,44,45]. By monitoring the amide H/D exchange process, we obtained extensive through-H-bond chemical shift isotope effects in the β-sheet, based on which the response of the surrounding H-bonds was constructed with the assistance of quantum mechanical (QM) calculations. If the weakening of the primary H-bond results in weakening (strengthens) of the surrounding H-bond, we define the two H-bonds as cooperative (anticooperative). By predicting the dihedral angle changes using QM calculations, we investigated the relationship between the structural adjustment and H-bond cooperativity.

## 2. Results

### 2.1. H/D Exchange Process in β-Sheet

**Figure 1:**
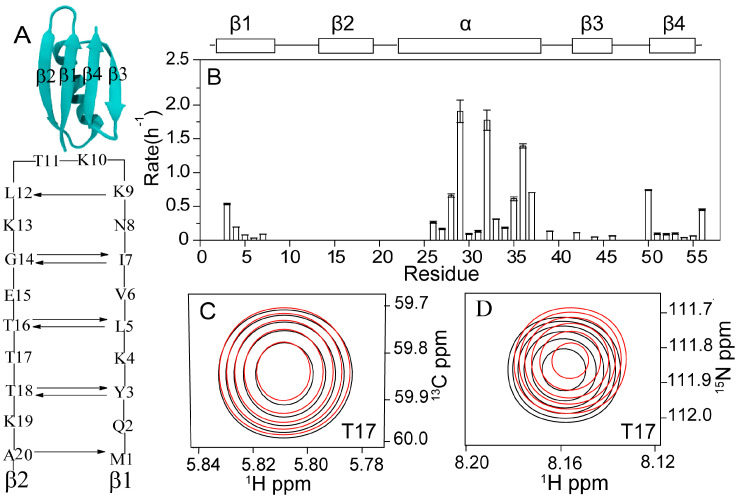
(**A**) H-bonding interactions between β1 and β2 of GB3. (**B**) H/D exchange rates versus residues of protein GB3 measured at pH 6.4, 298 K, in 100% D_2_O. The residues without rates have fast exchanges (*k*_ex_ > 5 h^−1^). The exchange rates are also listed in Table S1. Overlay of the first and last (**C**) ^1^H-^13^C and (**D**) ^1^H-^15^N HSQC spectra of residue T17 shows that its backbone chemical shifts of ^1^H_α_,^13^C_α_, ^1^H_N_, and ^15^N change in the H/D exchange process (black, the first spectrum measured at 0.5 h; red, the last spectrum measured at 12 h). Since T17 and all other residues in β2 have fast H/D exchanges, these chemical shift changes are due to the H/D exchange of the amides in β1. The profiles of the chemical shift changes are shown in Figure 2.

By dissolving the protonated ^15^N/^13^C labeled GB3 in 100% D_2_O (10 mM sodium phosphate, pH 6.4, 298 K), the H/D exchange rates were measured using the ^1^H-^15^N HSQC experiment (Table S1). The experimental dead time (time interval between the sample mixing with the buffer and the start of the first NMR spectrum acquisition) was 30 min. A 15-min spectrum of ^1^H-^15^N HSQC was recorded, followed by a 45-min ^1^H-^13^C HSQC spectrum. A total of 12 ^1^H-^15^N HSQC and ^1^H-^13^C HSQC spectra were recorded in 12 h. As can be seen, the amides in the second β-strand (β2, K13–K19) have fast exchanges beyond the detection limit (with the exchange rate *k*_ex_ > 5 h^−1^), although the N–Hs of G14, T16, and T18 are H-bonded to the C=Os of I7, L5, and Y3, respectively (Figure 1A,B). In contrast, the N–Hs of Y3, L5, and I7, which are H-bonded to the C=Os of T18, T16, and G14, respectively, have detectable exchange rates. The strand β1 (M1–N8) aligns antiparallel with β2, and seven H-bonds are formed in between (Figure 1A). The other side of the β2 is directly exposed to the solvent. The fast exchange rate of β2 indicates that the chemical shift change of the residues in this strand is caused by the H/D exchange of backbone amides not in β2 but in β1. For example, overlay of the first and the last (Figure 1C) ^1^H-^13^C and (Figure 1D) ^1^H-^15^N HSQC spectrum of residue T17 shows that its backbone chemical shifts of ^1^H_α_,^13^C_α_, ^1^H_N_, and ^15^N change in the H/D exchange process. Since T17 and all other residues in β2 have fast H/D exchange rates (already done before the first NMR spectrum was recorded completely), these chemical shift changes are due to H/D exchange of the amides in β1.

**Figure 2:**
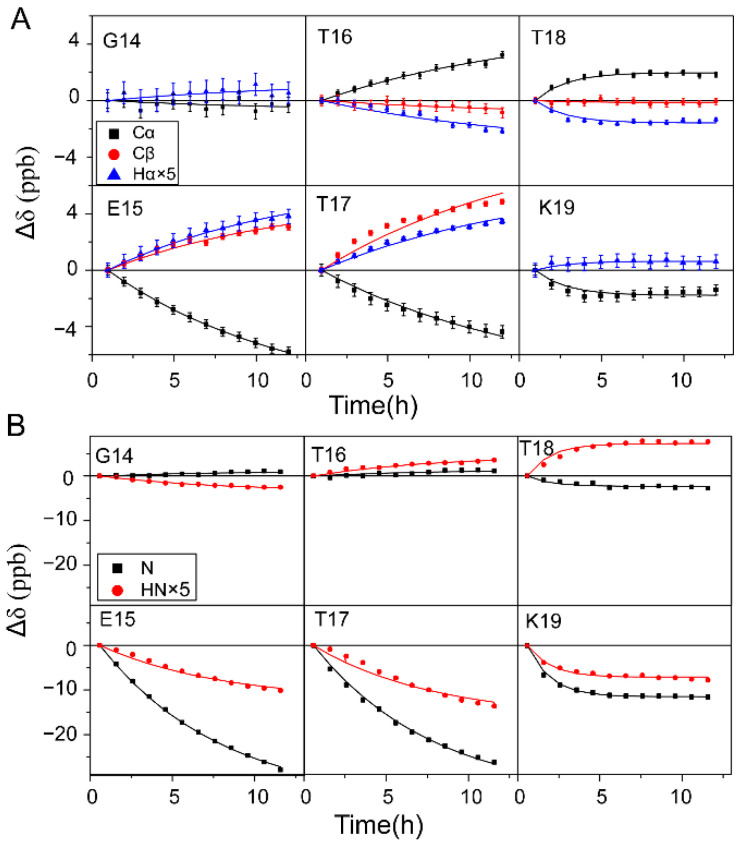
Chemical shift changes Δ*δ*s (*δ_t_* − *δ*_*t*0_) of ^1^H_α_, ^13^C_α_, and ^13^C_β_ (**A**) and ^1^H_N_ and ^15^N (**B**) as a function of H/D exchange time. The magnitude of ^1^H_α_ and ^1^H_N_ Δ*δ* is multiplied by a factor of 5 to give a better view. Δ*δ*s of residues G14 (E15), T16 (T17), and T18 (K19) were fitted to the H/D exchange rates of I7, L5, and Y3, respectively, using Equation (1). The C_β_ chemical shift of K19 cannot be retrieved due to signal overlap. The error bars are from duplicate experiments. The Δ*δ*s of all other residues are shown in Figures S2 and S3.

The ^1^H_α_, ^13^C_α_, and ^13^C_β_ chemical shift changes Δ*δ* (Δ*δ* = *δ_t_* − *δ*_*t*0_, where *δ_t_* and *δ*_*t*0_ are the chemical shifts at time *t* and *t*_0_, respectively) were recorded with a series of ^1^H-^13^C HSQC experiments (the resonance assignments for GB3-E27Q residues are illustrated in Figure S1), and those of residues G14–K19 are shown (Figure 2A, scatters in the figure). For residues T16 and T18, Δ*δ* of ^13^C_α_ increases but Δ*δ* of ^1^H_α_ decreases, whereas Δ*δ* of ^13^C_β_ is quite small. In contrast, for residues E15, T17, and K19, Δ*δ* of ^13^C_α_ decreases but that of ^1^H_α_ and ^13^C_β_ increases (^13^C_β_ Δ*δ* of K19 is not available due to signal overlap, Figure S1). For G14, Δ*δ* of ^13^C_α_ (^1^H_α_) decreases (increases) slightly. It is known that ^1^H_α_, ^13^C_α_, and ^13^C_β_ chemical shifts are sensitive to the backbone (*ϕ, ψ*) dihedral angles [46]. The change of these chemical shifts indicates that the (*ϕ, ψ*) angles of G14–K19 are perturbed. 100% D_2_O was used in the experiments to remove the overlap between water ^1^H and protein ^1^H_α_ signals at the same frequency. One caveat of using 100% D_2_O is that the [^1^H, ^15^N] signals of amides disappear after the H/D exchange is completed. That means one cannot follow ^1^H_N_ and ^15^N chemical shift changes for fast-exchanged amides. To solve this problem, the change of amide ^1^H and ^15^N chemical shifts was observed by recording ^1^H-^15^N HSQC spectra for the ^15^N/^13^C labeled GB3 dissolved in 70% D_2_O. For residues E15, T17, and K19, Δ*δ* decreases for both ^1^H_N_ and ^15^N whereas for residues G14, T16 and T18, Δ*δ* is generally smaller and different profiles are shown (Figure 2B, scatters in the figure). It is known that ^1^H_N_ and ^15^N chemical shifts are sensitive to the H-bond strength change of the exchanging amide group, which impacts the electron distribution around the observed backbone amide nuclei. The change of these chemical shifts indicates that the strength of the H-bond formed by G14–K19 is perturbed. Meanwhile, chemical shift changes Δ*δ* of ^1^H_α_, ^13^C_α_, ^13^C_β_, ^1^H_N_, and ^15^N of other residues as a function of H/D exchange time are shown in Figures S2 and S3. In summary, as the H/D exchange proceeds for residues in β1, the chemical shifts change for residues in β2, indicating the perturbation of structure and H-bond strength around the β2 residues. The relationship between them will be discussed below.

### 2.2. The Isotope Effect on Chemical Shifts Obtained from the NMR H/D Exchange Measurement

In order to investigate the relationship between the chemical shift change of residues in β2 and the H/D exchange process, the Δ*δ*s of each nucleus can in principle be fitted to a multi-exponential equation [10]. However, if there is only one H/D exchange site that contributes to Δ*δ*s, a single exponential fit is sufficient (see discussion below),

(1) $$\Delta \delta \left(t\right)=-c\lambda \left(exp\left(-kt\right)-exp\left(-kt_{0}\right)\right)$$

where *c* is the fraction of D_2_O in the solution, *k* is the exchange rate of the backbone amide site that causes Δ*δ*, *t*_0_ and *t* are the midpoints of the first and subsequent ^1^H-^13^C (or ^1^H-^15^N) HSQC measurements, *λ* is the chemical shift change contributed by the H/D exchange. The exchange rate of each amide was determined by the exponential decay of the peak intensity in the ^1^H-^15^N HSQC spectrum. So each amide has its own exchange rate. For the same peak in the spectrum, its position also changes at different time points (Figure 1D and Figure 2B), which is caused by H/D exchanges of the surrounding amides (not by its own H/D exchange, which only decreases its peak intensity). In the simplest case, we can attribute the chemical shift change to the H/D exchange of one particular surrounding amide (Equation (1)). So *λ* is the only parameter in the fitting of Equation (1), since the exchange rate *k* is already known. Ambiguity arises when the exchanges at multiple sites contribute to Δ*δ* (corresponding to a multi-exponential fitting). Fortunately, for the β1–β2 H-bond network, the fitting appears to be simple. For example, for K19, the Δ*δ*s of ^1^H_α_, ^13^C_α_, ^1^H and ^15^N have a rather fast decay profile, which fits well by using the exchange rate of Y3 solely (Figure 2). The exchange rates of K4, L5, V6, and I7 are too slow for the fitting of Δ*δ*s to K19. Similarly, the T18′s Δ*δ*s can be well fitted by using the Y3 exchange rate alone. The importance of Y3 H/D exchange to Δ*δ* of T18 and K19 can be rationalized structurally through the H-bond between N–H of Y3 and C=O of T18 (Figure 1A). The exchange of Y3 N–H to N–D weakens this H-bond and perturbs the backbone structure of T18 and K19. Similarly, Δ*δ*s of G14 and T15, and T16 and T17, can be fitted using the exchange rate of their H-bond partners I7 and L5, respectively (Figure 2). It is worth mentioning that the backbone amides in β2 have completed H/D exchanges within 30 min. Therefore, the isotope effect from the H/D exchanges of precedent and subsequent amides (the through-bond effect) was not observable, which greatly simplified the data analysis. However, for residues in β3 and β4, the fitting ambiguity cannot be resolved because residues in both strands have observable and somewhat comparable exchange rates (Figure 1), and thus *λ* values cannot be reliably retrieved.

**Figure 3:**
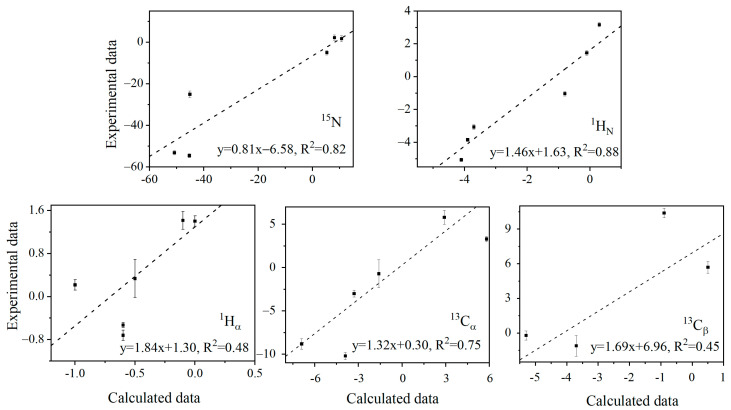
Correlation of *λ* values from the experimental fitting of Δ*δ*s to Equation (1) for different nuclei and from the ONIOM/DFT calculations.

The distinctive pattern of ^13^C_α_, ^13^C_β_, and ^1^H_α_ *λ* values suggests that the (*ϕ*, *ψ*) changing direction of E15, T17, and K19 is similar, opposite to that of T16 and T18 (Table S2). However, deriving the protein structural reorganization directly from *λ* values is not straightforward. Here, QM ONIOM calculations [47] were performed to predict *λ* values. The anharmonic N–H bond stretch vibration elongates the bond length due to the cubic nature of the potential energy. N–D has a smaller bond length elongation than N–H because D has a heavier atomic mass than H. The cubic average bond lengths of N–H and N–D of Y3, L5, and I7, derived from ONIOM calculations, are listed in Table 1. The shorter cubic average N–D bond length corresponds to a longer D–O distance [43] and thus a weaker N–H(D)…O=C H-bond strength [43,48]. The chemical shift *δ* of each nucleus was calculated using b3lyp/6-31++g(d,p) for the two optimized structures, with the N–H (D) bond length fixed at the corresponding cubic average value of N–H (D). The correlations of *λ* values for different nuclei are shown in Figure 3 between the b3lyp predicted and experimental data. For the amides ^15^N, ^1^H_N_, and ^13^C_α_, the QM calculations predict *λ* reasonably well (R^2^ = 0.82, 0.88, and 0.75). But for ^13^C_β_ and ^1^H_α_ of G14, E15, T17, and K19, the correlation is moderate (R^2^ = 0.45, 0.48). H_α_ and C_β_ are further away from the H-bond. It becomes harder to capture their minute structure change caused by H-bond perturbation. This is also consistent with the fact that the slope is larger for these two nuclei, meaning that the predicted variation of lambda values is smaller (relative to that from the experiment) than for the other three nuclei. Considering that the geometric change caused by the H/D exchange is notoriously small (see discussion below), in addition to the influence of amino acid side chains or surrounding water molecules [49,50], it is extremely challenging to reproduce the experimental *λ* values computationally, particularly for those away from amide ^1^H_N_. Nevertheless, the semi-quantitative agreement with the experimental data permits us to look further into H-bond cooperativity.

## 3. Discussion

**Figure 4:**
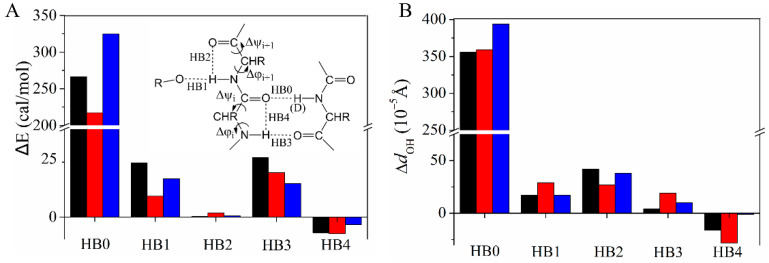
Changes of the H-bond energy Δ*E* (Δ*E* = *E*(D) − *E*(H) (**A**) and the H-bond distance between H of the donor and O of the acceptor (**B**) when substituting N–H by N–D for Y3 (black), L5 (red), or I7 (blue). HB0 and HB3 are between β1 and β2 whereas HB2 and HB4 (so called “C5 H-bond”) are within β2. HB1 is the H-bond formed between the amide and the side chain (NH of T17 with O_γ_H of T16; NH of K19 with O_γ_H of T18) or water (NH of E15 with OH of water) (Figures S5–S7).

The QM calculations indicate that the substitution of a backbone N–H by N–D affects the (*ϕ*, *ψ*) dihedral angles of its H-bond partner (Table 2). Taking L5 as an example, the QM calculations predict that (*ϕ*, *ψ*) of T16 are changed by (−0.018°, −0.026°) whereas (*ϕ*, *ψ*) of T17 are changed by (0.032°, 0.002°) when the N–H of L5 is substituted by the N–D. The different changing directions for the neighboring (*ϕ*, *ψ*) pairs can be seen for the other two H-bond systems, Y3→T18 and I7→G14. The H/D exchange in the primary H-bond (named “HB0” in the Figure 4 insert) perturbs four neighboring H-bonds involving β2 (HB1–4, Figure 4). The natural bond orbital (NBO) analysis [51] was performed to quantify the energy difference of all five H-bonds (Δ*E* = *E*(D) − *E*(H), Figure 4A). HB0 is weakened as expected when H is substituted by D, whereas the surrounding H-bonds HB1, HB2, HB3, and HB4 have different responses. HB1, HB2, and HB3 become weaker, whereas HB4 becomes stronger. HB2 and HB4 are the so-called C5 H-bond which is important for β-sheet stability and sensitive to the backbone dihedral angles [52,53]. The perturbation of the H-bond is also reflected by the distance change between O of the acceptor and H_N_ of the donor, *d*_OH_. Although *d*_OH_ is very small, its changing pattern matches wth that of the NBO H-bond energy (Figure 4B). Moreover, the opposite signs of HB2 and HB4 energy changes are consistent with the opposite (*ϕ*, *ψ*) changes of residues *i* and *i* + 1 (Table 2). To further understand the importance of (*ϕ*, *ψ*) changes on the perturbation of the four H-bonds, control calculations were performed by only changing the primary N–H (D) bond distance while fixing all other degrees of freedom so that the (*ϕ*, *ψ*) angles were unchanged. ΔEs of HB1 and HB2 are very small (Figure S4), suggesting that the change of these two H-bonds is caused by (*ϕ*, *ψ*) structural reorganization. In comparison, the Δ*E*s of HB3 and HB4 are somewhat larger; they have the same signs as those in Figure 4. For these two H-bonds, the electron density redistribution on HB0 caused by the H/D exchange also perturbs their strengths.

There are a few limitations in the QM calculations. The main limitation is that the solvent effect was not properly included in the model. Solvent water creates a polar environment with a high dielectric constant, which is expected to affect protein H-bonds. But due to the dynamic nature of water molecules, it is almost impossible to add them properly in the calculations. The second limitation is that protein dynamics were missing. Proteins are not rigid, especially the side chains. In the DFT calculations, a single static structure was used. How the dynamics of proteins and solvents may affect computational results is hard to predict. One could imagine that a lot of noise from dynamics may obscure the structural changes, considering that the changes are so small. This will make the calculations impractical. But the correlation between experimental and computational *λ* suggests that using a single static structure is not a terrible choice (Figure 3).

The positive cooperativity between HB0 and HB1 has been suggested previously through the backbone amide proton chemical shift [54] and the across-H-bond *^3h^J_NC′_* constant [37]. This cooperativity helps explain the correlation of protein backbone dynamics across the β-sheet, which effectively restrains the motion of backbone amides [26]. The long-range propagation of amide out-of-plane vibrations observed from residual dipolar coupling experiments may also come from the cooperativity between HB0 and HB1 [55]. The same sign for HB1 and HB2 as that of HB0 indicates that strengthening HB0 will increase the strength of HB1 and HB2. Alternatively, when a β-strand is added and H-bonded to the β-sheet (through e.g., HB1), the existing β-sheet conformation is stabilized through strengthening of HB0. In contrast, HB3 and HB4 have opposite signs and essentially cancel out each other. The net effect is that strengthening or weakening HB0 has a minor effect on HB3 and HB4 together. These results explain the stability measurements of antiparallel β-sheets, in which adding strands perpendicular to the β-sheet stabilizes the folded conformation while extending the β-strand length can either stabilize or destabilize the folded conformation [56,57,58]. The complex nature of H-bond cooperativity is also expected to contribute to the distinctive nature of the correlated β-sheet conformational dynamics [59].

## 4. Materials and Methods

### 4.1. NMR Spectroscopy and Exchange Rate Fitting

The GB3 E27Q mutant expression and purification were previously described [60]. Final samples were prepared in a buffer consisting of 10 mM sodium phosphate at pH 6.4 in a 30%/70% (*v*/*v*) H_2_O/D_2_O mixture or 100% D_2_O. All NMR experiments were carried out at 298 K on a Bruker Avance 600-MHz spectrometer equipped with a *z*-axis gradient, triple resonance, and a cryogenic probe. For the ^1^H_α_, ^13^C_α_, and ^13^C_β_ chemical shifts measurements, 5 mg of ^15^N/^13^C-labeled protonated GB3 E27Q powder was added to a 500 μL 100% D_2_O sodium phosphate buffer, which was then transferred to a NMR tube and inserted into a NMR spectrometer. A series of interleaved 2D gradient-enhanced ^1^H-^15^N HSQC spectra [61] and 2D ^1^H-^13^C HSQC spectra with the Rance-Kay readout [61,62] were recorded to monitor the H/D exchange process. For the 2D ^1^H-^15^N HSQC, the acquisition times were 70.5 ms (^15^N) and 83.0 ms (^1^H) with data matrices of 94* × 1024* complex data points. For the 2D ^1^H-^13^C HSQC, the acquisition times were 24.0 ms (^13^C) and 72.6 ms (^1^H) with data matrices of 240* × 720* complex data points. It took 15 min and 45 min to record 2D ^1^H-^15^N and ^1^H-^13^C HSQC, respectively. Thus, one ^1^H-^15^N spectrum was recorded, followed by one ^1^H-^13^C HSQC spectrum, in each hour. The dead time for the H/D exchange was 0.5 h. A total of 12 spectra were recorded for both HSQCs over a period of 12.5 h. The H/D exchange experiment was performed twice, yielding an estimate of the exchange rate and chemical shift change errors.

For the ^1^H_N_ and ^15^N chemical shift measurements, a series of 2D ^1^H-^15^N HSQC were recorded for the ^15^N/^13^C-labeled protonated GB3 E27Q powder dissolved in 30%/70% H_2_O/D_2_O. It took 30 min to record one 2D spectrum. The acquisition parameters of the ^1^H-^15^N HSQC were the same as those measured for the 100% D_2_O sample, except that the number of scans was doubled. A total of 12 spectra were recorded for the 2D experiment in 12.5 h. The H/D exchange experiment was performed twice. All the NMR spectra were processed and analyzed using NMRPipe and NMRDraw [63].The exchange rates were obtained by fitting the 2D ^1^H-^15^N HSQC peak height of each residue to a two-parameter exponential function and scaling it down by a factor of 1.074 to correct for the fitting bias [10].

### 4.2. ONIOM Calculations

The H-bonding structures and surrounding residues were modeled using a three-layer QM ONIOM method (m062×/6-31++g(d,p):b3lyp/6-31++g(d,p):hf/sto-3g) [64]. The m062× DFT method is particularly accurate in describing the H-bonded system [65]. Three computational models were truncated from the X-ray structure of GB3 (PDB ID: 2OED). The first model Y3-T18, which describes the H-bond between Y3 and T18, includes residues M1, Q2, Y3, K4, L5, T16, T17, T18, K19, A20, V21, D22, A23, A26, A29, F30, Y45, D47, K50, T51, and F52 (Figure S5). The second model L5-T16, which describes the H-bond between L5 and T16, includes residues Q2, Y3, K4, L5, V6, I7, G14, E15, T16, T17, T18, K19, F30, Y33, A34, W43, Y45, T49, K50, T51, F52, T53, and V54 (Figure S6). In the structure optimization, the O_γ_H of T16 twisted the (*φ*, *ψ*) angles of T17 to form an artificial H-bond with the T17 C=O group. To prevent this, a water molecule was added in between, and the model was then reoptimized, yielding a geometry that agreed better with the X-ray structure. The third model I7-G14, which describes the H-bond between I7 and G14, includes residues K4, L5, V6, I7, N8, G9, L12, K13, G14, E15, T16, T17, Y33, V39, T51, F52, T53, and V54 (Figure S7). The X-ray structure shows that no H-bond is formed with the E15 amide N–H. However, the E15 amide ^1^H chemical shift of 8.417 ppm is comparable to that of G14 (8.298 ppm), T16 (8.792 ppm), and other β-sheet residues that are H-bonded, suggesting that the amide of E15 is H-bonded to solvent water [66]. Two water molecules were added to the model to form the putative H-bond (Figure S7). The details of atoms treated at the high, middle, and low levels are shown in Figure S5B, Figure S6B and Figure S7B, respectively. All the charged residues were neutralized in the calculations, which were performed in the gas phase.

After the optimization, the N–H bond length of Y3, L5, and I7 in the three models was scanned with a distance interval of 0.02 Å, yielding a potential energy curve from *d* − 0.1 Å to *d* + 0.1 Å where *d* is the equilibrium bond length. The average bond length of N–H (*r*_H_) and N–D (*r*_D_) was derived from the curve using the method described in the literature [67] and listed in Table 1. By constraining the N–H length of Y3, L5, or I7 to the calculated *r*_H_ or *r*_D_, the models were reoptimized to yield the H or D form. Then, the low-level residues were removed, whereas the NMR shielding parameters were calculated at b3lyp/6-31++g(d,p) for the high and middle-level residues. The b3lyp DFT has been widely used in calculating chemical shifts and is known to yield good results with a reasonable computational cost [68]. The H/D isotope effect *λ* was obtained by *δ*(D) − *δ*(H) where *δ*(D) and *δ*(H) are the chemical shift of a nucleus in the D or H form. The accuracy of b3lyp on isotope effect calculations was tested on a H-bonded tri-N-methylacetamide ((NMA)_3_) complex by varying the primary H-bond lengths and compared with the MP2 calculations (Figure S8). A good correlation was observed between Δ*δ*s from the two methods, although b3lyp can slightly overestimate or underestimate the values depending on the type of nuclei. In addition, the natural bond orbitals (NBO) analyses were carried out at b3lyp/6-31++g(d,p), and the Fock matrix elements were used to evaluate the interactions between the hydrogen bond donor and acceptor orbitals [51]. The energy difference between the D and H forms was obtained by *E*(D) − *E*(H). All calculations were carried out using the Gaussian 09 program [69].

## 5. Conclusions

In summary, the H-bond cooperativity in β-sheets of an intact protein is studied by the H/D isotope effect. The weakening of the GB3 β1→β2 H-bond perturbs the intra- and inter-strand H-bonds, suggesting that these H-bonds are cooperative or anticooperative. The β-sheet is stabilized by the cooperativity/anticooperativity between HB0 and the other four H-bonds. Coupled backbone amide librational motion across the β-sheet of GB1 and GB3 has been demonstrated [26,55]. Our study demonstrates the relationship between the structural adjustment and hydrogen bond cooperativity, providing new evidence for understanding the interconnectivity of hydrogen bonding in the β-sheet of GB3.

## Figures and Tables

**Table 1:** Average bond lengths *^a^* of N–H and N–D.

	N–H (Å)	N–D (Å)
Y3	1.03398	1.02978
L5	1.03754	1.03324
I7	1.03473	1.03038

*^a^* The average bond length is the equilibrium bond length at the lowest energy plus the anharmonic bond stretching correction.

**Table 2:** Predicted changes of backbone *φ* and *ψ* dihedral angles of residues in β2 caused by the backbone amide H/D exchange in β1.

Exchange Site	∆*φ_i_^a^* (°)	∆*ψ_i_* (°)	∆*φ*_*i*+1_ (°)	∆*ψ*_*i*+1_ (°)
Y3→T18	−0.017	−0.026	0.044	0.008
L5→T16	−0.018	−0.026	0.026	0.001
I7→G14	−0.014	−0.020	0.019	0.004

*^a^ i* represents residues T18, T16, and G14 for Y3, L5, and I7, respectively (Figure 4). ∆*φ* = *φ*(D) − *φ*(H); ∆*ψ* = *ψ*(D) − *ψ*(H).

## Data Availability

The original contributions presented in the study are included in the article. Further inquiries can be directed to the corresponding author.

## Supplementary Materials

The following supporting information can be downloaded at: https://www.mdpi.com/article/10.3390/ijms232314821/s1, Table S1: H/D exchange rate measured at 298 K and a pH of 6.4 in 100% D_2_O or a 70%/30% D_2_O/H_2_O mixed solution; Table S2: *λ* values from the experimental fitting of Δ*δ*s to Equation (1) for different nuclei and from the ONIOM/DFT calculations; Figure S1: Resonance assignments of GB3-E27Q; Figure S2: Chemical shift changes Δ*δ* (*δ_t_* − *δ*_*t*0_) of ^1^H_α_, ^13^C_α_, and ^13^C_β_ of different residues as a function of H/D exchange time; Figure S3: Chemical shift changes Δ*δ* (*δ_t_* − *δ*_*t*0_) of ^1^H_N_ and ^15^N of different residues as a function of H/D exchange time; Figure S4: H-bond energy changes Δ*E* (Δ*E* = *E*(D) − *E*(H)); Figure S5: H-bond model Y3-T18 built for the ONIOM calculation; Figure S6: H-bond model L5-T16 built for the ONIOM calculation; Figure S7: H-bond model I7-G14 built for the ONIOM calculation. Figure S8: Benchmark of B3LYP calculated Δ*δ*s against the MP2/6-311+g* results.
