# Supplementary material for: Detecting the Hydrogen Bond Cooperativity in a Protein β-Sheet by H/D Exchange
Source: Int J Mol Sci. 2022 Nov 26;23(23):14821. doi: 10.3390/ijms232314821 (PMC9740688; doi:10.3390/ijms232314821)
Supplement: Supplementary file 1 [file ijms-23-14821-s001.zip › ijms-2023090-supplementary.pdf]

Supporting Information

Detecting the Hydrogen Bond Cooperativity in a Protein β-sheet by H/D Exchange

Jingwen Li [1], Jingfei Chen [2, 3], Yefei Wang [2, 3, *] and Lishan Yao [2,3, *]

[1] College of Chemistry and Chemical Engineering, China University of Petroleum (East China), Qingdao 266580, China

[2] Qingdao New Energy Shandong Laboratory, Qingdao Institute of Bioenergy and Bioprocess Technology, Chinese Academy of Sciences, Qingdao 266101, China.

[3] Shandong Energy Institute, Qingdao 266101, China.

* Correspondence: wangyf@qibebt.ac.cn (Y.W.); yaols@qibebt.ac.cn (L.Y.)

Table S1. H/D exchange rates measured at 298 K and pH of 6.4 in 100% D_2O or 70%/30% D_2O/H_2O mixed solution.

Res	Rate (h^{-1}) 100% D_2O	Rate (h^{-1}) 70% D_2O
2	f[a]	f
3	0.536±0.011	0.71±0.023
4	0.196±0.001	0.351±0.013
5	0.076±0.001	0.136±0.005
6	0.032±0.002	0.053±0.001
7	0.088±0.002	0.135±0.003
8	f	f
9	f	f
10	f	f
11	f	f
12	f	f
13	f	f
14	f	f
15	f	f
16	f	f
17	f	f
18	f	f
19	f	f
20	f	f
21	f	f
22	f	f
23	f	f
24	f	f
25	f	f
26	0.266±0.015	0.408±0.005
27	0.167±0.012	0.274±0.008
28	0.660±0.026	0.848±0.037
29	1.906±0.167	2.078±0.207
30	0.092±0.009	0.166±0.005
31	0.131±0.013	0.235±0.008
32	1.775±0.151	2.062±0.175
33	0.312±0.005	0.422±0.005
34	0.184±0.011	0.305±0.010
35	0.613±0.027	0.934±0.045
36	1.392±0.032	1.66±0.127
37	0.705±0.001	0.84±0.027
38	f	f

39	0.134±0.002	0.175±0.005
40	f	f
41	f	f
42	0.111±0.003	0.138±0.003
43	f	f
44	0.047±0.003	0.084±0.003
45	f	f
46	0.065±0.005	0.108±0.003
47	f	f
48	f	f
49	f	f
50	0.740±0.007	0.84±0.045
51	0.096±0.011	0.175±0.008
52	0.087±0.011	0.165±0.007
53	0.098±0.013	0.184±0.009
54	0.039±0.003	0.065±0.002
55	0.062±0.003	0.097±0.003
56	0.451±0.013	0.498±0.002

[a.] The exchange rate is faster than ~ 5 h^{-1}.

Table S2. λ values from the experimental fitting of $\Delta\delta$s to eq. 1 for different nuclei and from the ONIOM/DFT calculations

	N		H_N		C_α		C_β		H_α	
	Ex[b]	Ca[c]	Ex	Ca	Ex	Ca	Ex	Ca	Ex	Ca
14	1.7 ±1.4	10.7	−1.04 ±0.13	−0.8	−0.7 ±1.6	−1.6	N[d]	N	0.34 ±0.35	− 0.5
15	-54.5 ±0.9	−45.3	−3.84 ±0.11	−3.9	−10.2 ±0.4	−3.9	5.7 ±0.5	0.5	1.42 ±0.16	−0.1
16	2.2 ±1.3	8.1	1.45 ±0.15	−0.1	5.8 ±0.8	2.9	-1.1 ±0.9	-3.7	-0.72 ±0.10	−0.6
17	−53.1 ±1	−50.8	−5.06 ±0.09	−4.1	−8.8 ±0.6	−6.9	10.4 ±0.4	-0.9	1.40 ±0.09	0
18	−5 ±1.1	5.3	3.17 ±0.13	0.3	3.3 ±0.3	5.8	-0.2 ±0.4	-5.3	-0.53 ±0.04	−0.6
19	−25 ±1.6	−45.1	−3.06 ±0.14	−3.7	−3 ±0.4	−3.3	N	N	0.22 ±0.1	−1

[b.] "Ex": Experimental data

[c.] "Ca": Calculated data

[d.] No data

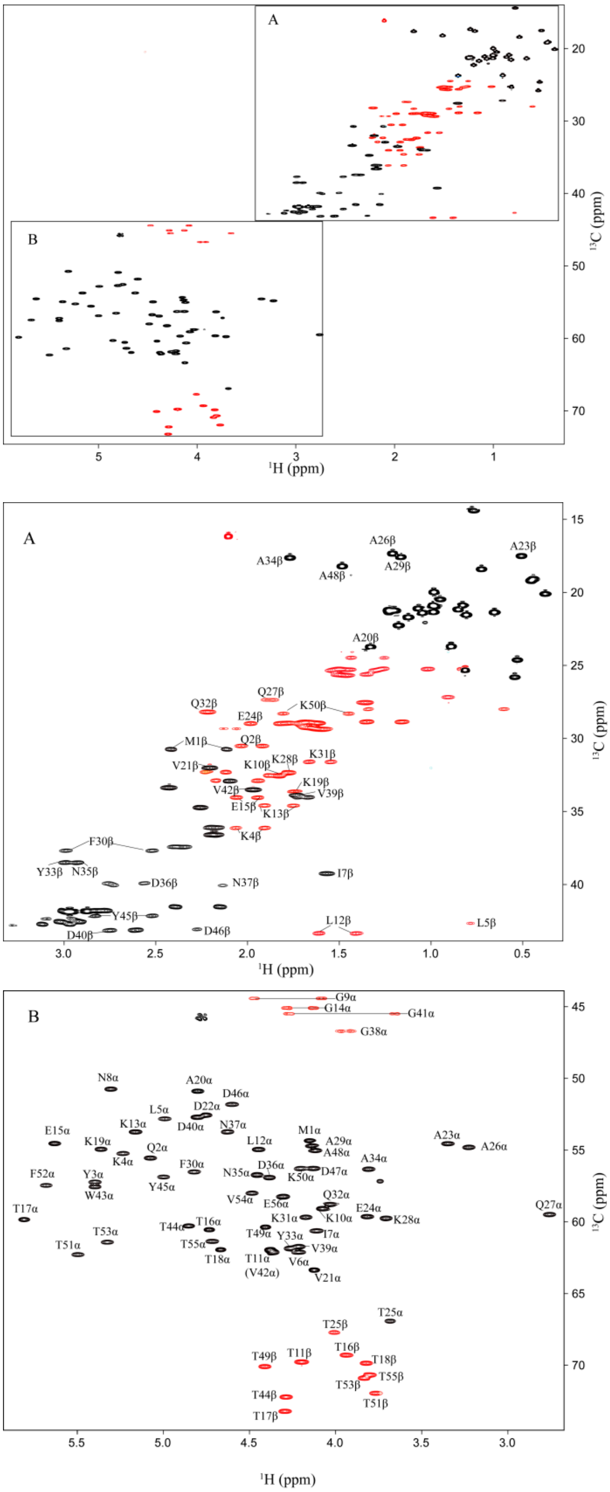

Figure S1. Resonance assignments of GB3-E27Q. The first ^{1}H-^{13}C HSQC spectrum of H/D exchange at 298K, pH6.4, 100%D_2O.

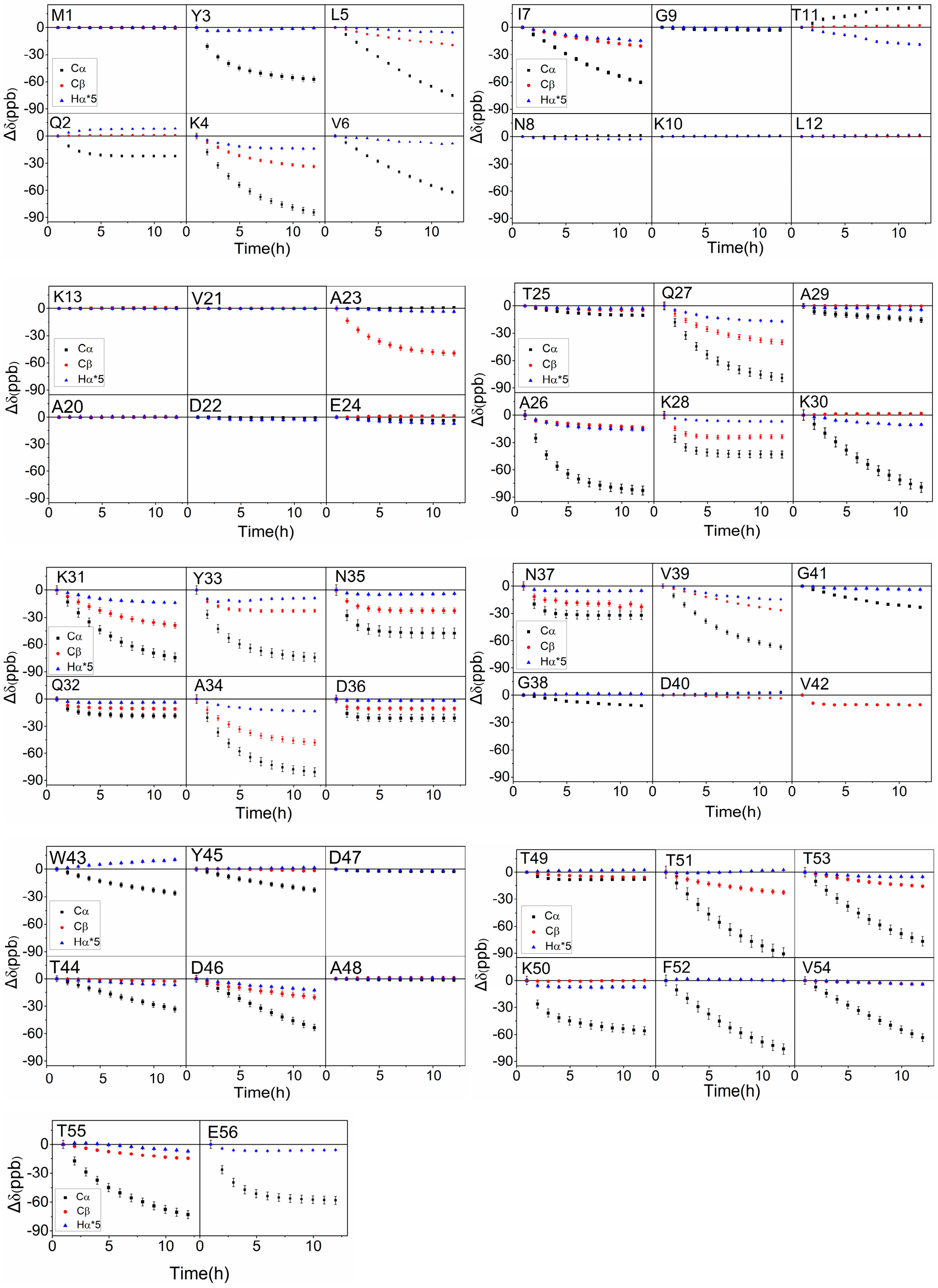

Figure S2. Chemical shift changes $\Delta\delta$ ($\delta_t - \delta_{t0}$) of $^1H_\alpha$, $^{13}C_\alpha$, and $^{13}C_\beta$ of different residues as a function of H/D exchange time. Residues of 14–19 are shown in Figure 2A.

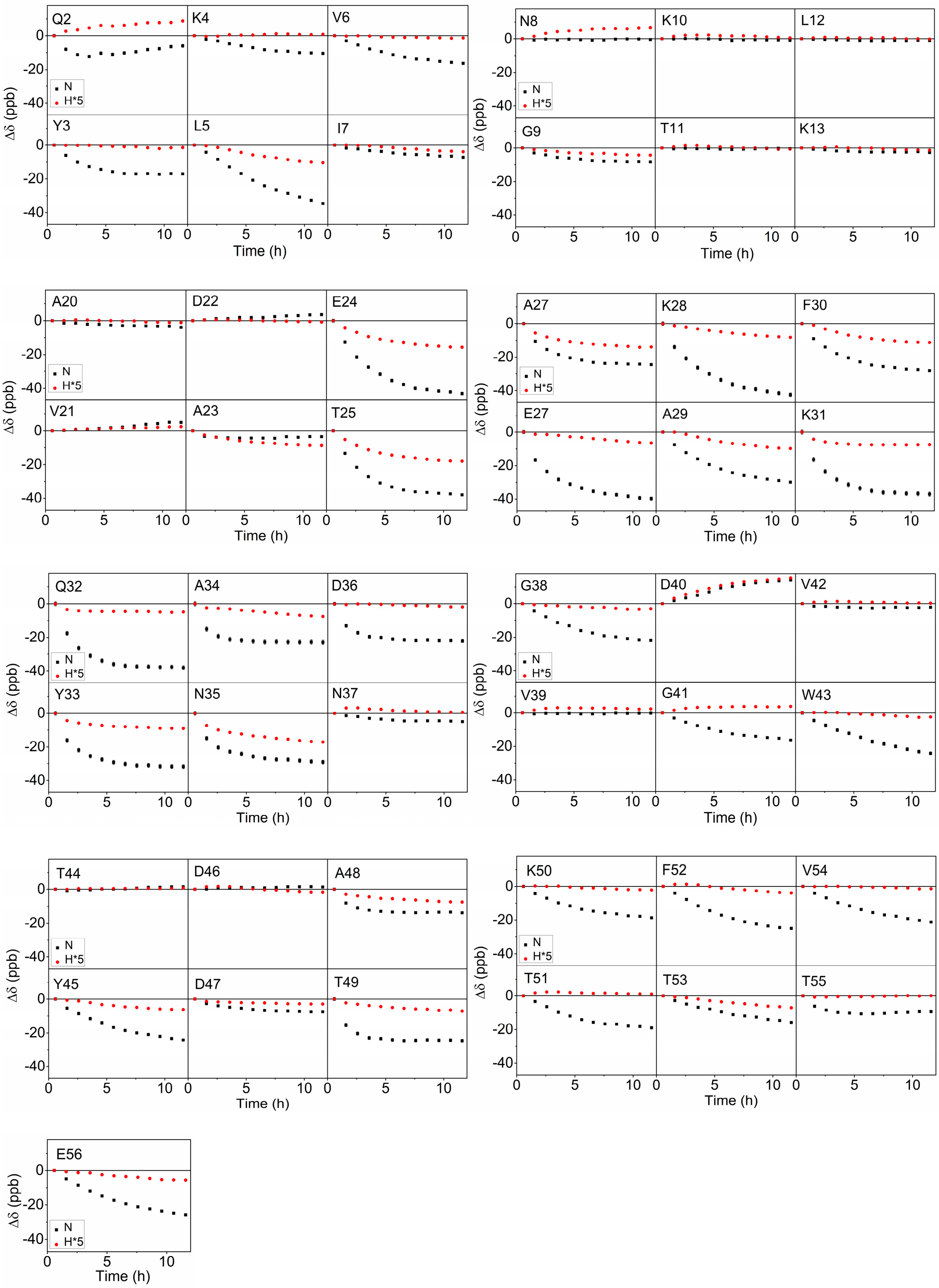

Figure S3. Chemical shift changes $\Delta\delta(\delta_t - \delta_{t0})$ of ^1H_N and ^{15}N of different residues as a function of H/D exchange time. Residues of 14–19 are shown in Figure 2B.

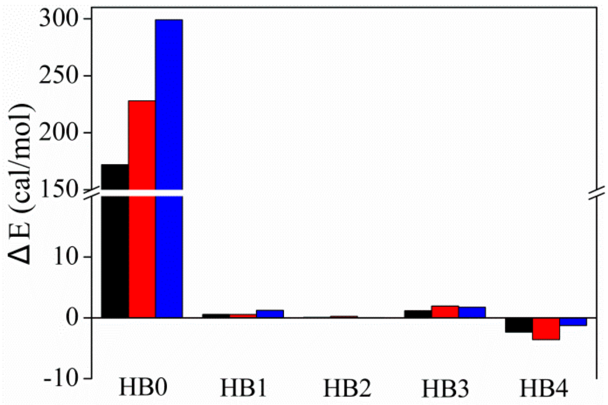

Figure S4. H-bond energy changes Δ*E* (Δ*E* = *E*(D) – *E*(H)) when substituting N–H by N–D for Y3 (black), L5 (red), or I7 (blue) by only changing the N–H (D) bond distance while fixing all other degrees of freedom. HB0–HB4 are defined in Figure 4.

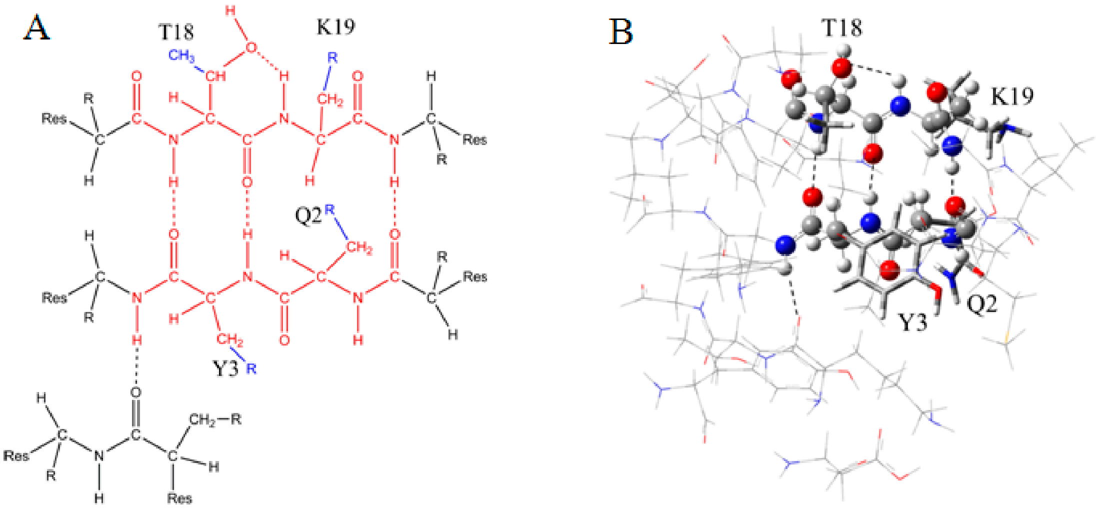

Figure S5. H-bond model Y3-T18 built for the ONIOM calculation. **(A)** Schematic drawing of the model. The atoms treated in the high and middle levels are shown in red and blue respectively. The atoms treated in the low level are shown in black. For the sake of clarity, certain residues in the low level are not included but listed in the main text of the supporting information. **(B)** Structure after the ONIOM optimization. The atoms in the high, middle, and low levels are shown by ball and stick, stick, and line, respectively.

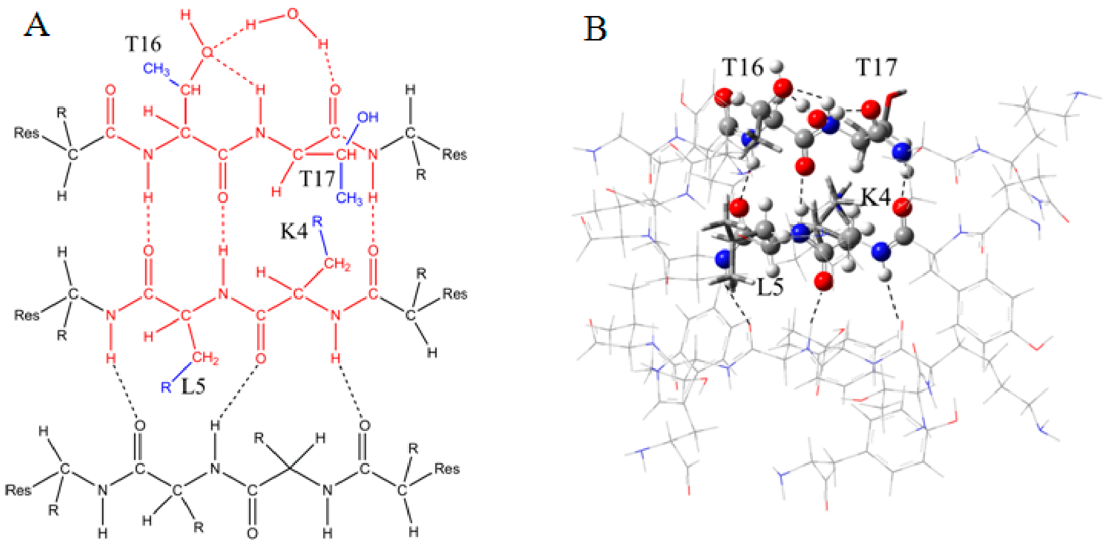

Figure S6. H-bond model L5-T16 built for the ONIOM calculation. **(A)** Schematic drawing of the model. The atoms treated in the high and middle levels are shown in red and blue respectively. The atoms treated in the low level are shown in black. For the sake of clarity, certain residues in the low level are not included but listed in the main text of the supporting information. **(B)** Structure after the ONIOM optimization. The atoms in the high, middle, and low levels are shown by ball and stick, stick, and line, respectively.

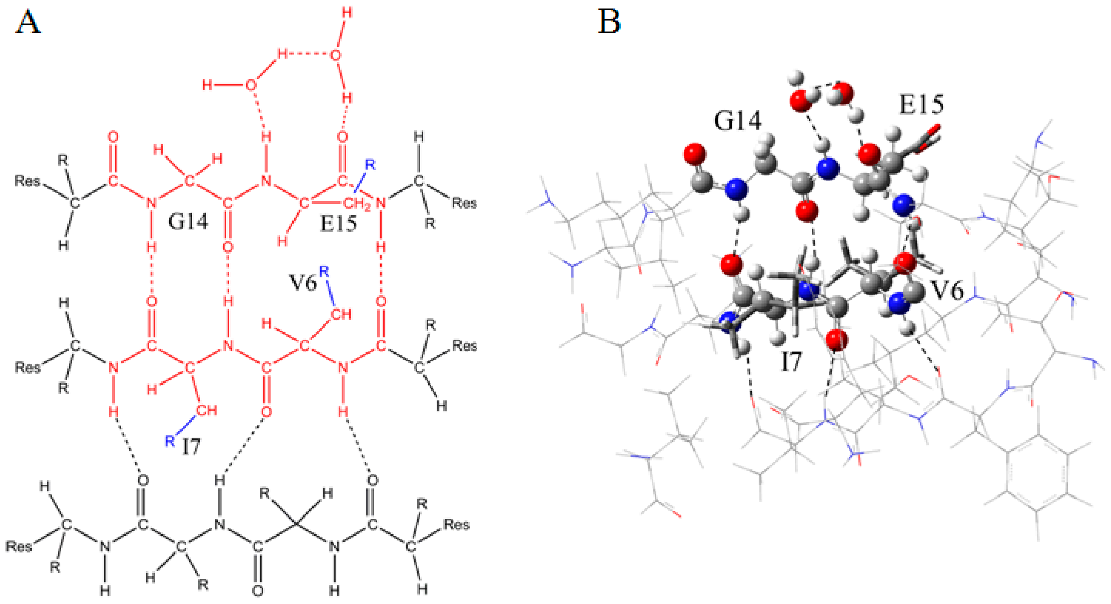

Figure S7. H-bond model I7-G14 built for the ONIOM calculation. **(A)** Schematic drawing of the model. The atoms treated in the high and middle levels are shown in red and blue respectively. The atoms treated in the low level are shown in black. For the sake of clarity, certain residues in the low level are not included but listed in the main text of the supporting information. **(B)** Structure after the ONIOM optimization. The atoms in the high, middle, and low levels are shown by ball and stick, stick, and line, respectively.

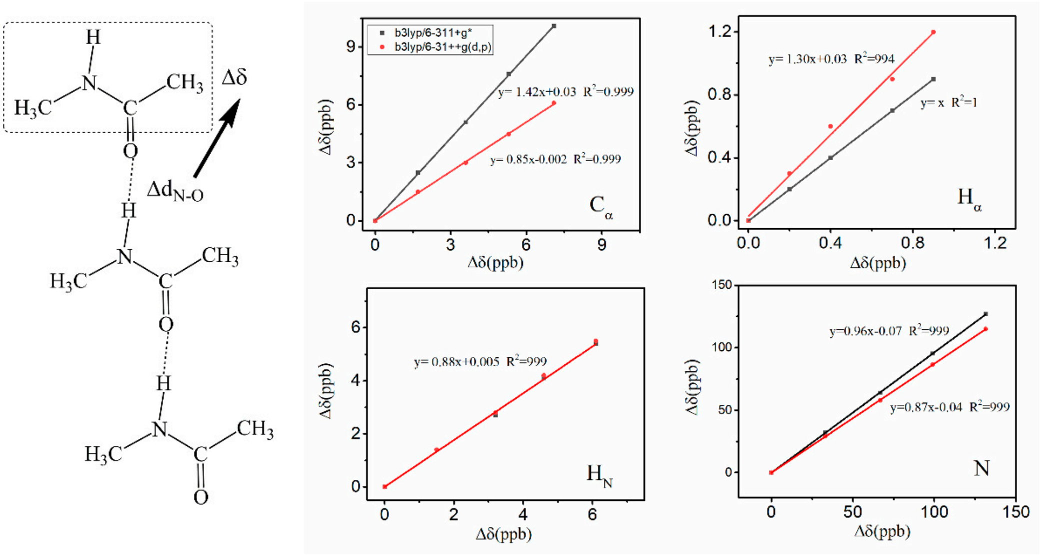

Figure S8. Benchmark of B3LYP calculated Δδs against the MP2/6-311+g* results. The $(NMA)_3$ was optimized and the distance $\Delta d_{N\text{-}O}$ was scanned from 0 (at the equilibrium distance) to 0.02 Å. The corresponding chemical shift changes Δδs were calculated using B3LYP or MP2 methods.
